# 2383. Immunogenicity and reactogenicity of a first booster with BNT162b2 or full-dose mRNA-1273: a randomised trial in adults ≥75 years (EU-COVAT-1) from the VACCELERATE consortium

**DOI:** 10.1093/ofid/ofad500.2003

**Published:** 2023-11-27

**Authors:** Jon Salmanton-Garcia, Julia M Neuhann, Jannik Stemler, Antonio Carcas, Jesús Frías-Iniesta, Ullrich Bethe, Sarah Heringer, Lea Tischmann, Marouan Zarrouk, Arnd Cuepers, Philipp Koehler, Jan H Grothe, Samir Kumar Singh, Alejandro García-León, Katherine Loens, Christine Lammens, Franz Koenig, Lusine Yeghiazaryan, Martin Posch, Oliver A Cornely

**Affiliations:** University Hospital Cologne, Cologne, Germany, Cologne, Nordrhein-Westfalen, Germany; University Hospital Cologne, Cologne, Nordrhein-Westfalen, Germany; University Hospital Cologne, Cologne, Nordrhein-Westfalen, Germany; Hospital Universitario La Paz, Madrid, Madrid, Spain; Hospital Universitario La Paz, Madrid, Madrid, Spain; University Hospital Cologne, Cologne, Nordrhein-Westfalen, Germany; University Hospital Cologne, Cologne, Nordrhein-Westfalen, Germany; University Hospital Cologne, Cologne, Nordrhein-Westfalen, Germany; University Hospital of Cologne, Cologne, Nordrhein-Westfalen, Germany; University Hospital Cologne, Cologne, Nordrhein-Westfalen, Germany; University Hospital Cologne, Cologne, Germany, Cologne, Nordrhein-Westfalen, Germany; University Hospital Cologne, Cologne, Nordrhein-Westfalen, Germany; University of Antwerp, Antwerp, Antwerpen, Belgium; Centre for Experimental Pathogen Host Research (CEPHR), University College Dublin, Belfield, Dublin 4, Ireland, Dublin, Dublin, Ireland; University of Antwerp, Antwerp, Antwerpen, Belgium; University Antwerp, Department of Medical Microbiology, Antwerp, Antwerpen, Belgium; Medical University Vienna, Vienna, Wien, Austria; Medical University Vienna, Vienna, Wien, Austria; Medical University Vienna, Vienna, Wien, Austria; University of Cologne, Cologne, Germany, Cologne, Nordrhein-Westfalen, Germany

## Abstract

**Background:**

Vaccination remains crucial for protection against severe SARS-CoV-2 infection, especially in aged population.

**Methods:**

We evaluated, with a randomised controlled, adaptive, multicentre phase II study, the safety and immunogenicity of a 3rd vaccination dose (1st booster) in individuals ≥75 years (ClinicalTrials.gov Identifier: NCT05160766, EudraCT Number: 2021-004526-29). Participants were randomized to either a full-dose (100µg) of mRNA-1273 (Spikevax^®^) or 30 µg of BNT162b2 (Comirnaty^®^). The primary endpoint was the rate of 2-fold antibody titre increase 14 days post-vaccination measured by quantitative enzyme-linked immunosorbent assay (Anti-RBD-ELISA). Secondary endpoints included changes in neutralising capacity (ACE2 Virus Neutralisation Assay) against wild-type and 25 variants at 14 days and up to 12 months.

**Results:**

Fifty-three volunteers were randomised between 8 November 2021 and 4 January 2022, with 52 receiving a COVID-19 vaccine as 1st booster. Fifty subjects (BNT162b2 n=25/mRNA-1273 n=25) were included in the analyses for immunogenicity after day 14. The primary endpoint of a 2-fold anti-RBD IgG titre increase 14 days post-vaccination was reached in all subjects. A 3rd full-dose mRNA-1273 vaccination provided higher anti-RBD IgG titres (GMT D14 7090 [95% CI 5688 – 8837] BNT162b2 vs. 10711 [95% CI 8003 – 14336] mRNA-1273). A pattern showing higher neutralising capacity of full-dose mRNA-1273 against Wuhan wild-type so as for 23/25 tested variants was observed.

**Conclusion:**

Third doses of either BNT162b2 or mRNA-1273 provide substantial antibody increase 14 days post-vaccination, with full-dose mRNA providing higher antibody levels and an overall similar safety profile for ≥75 subjects. Additional booster doses should be prioritised, particularly in aged people and others at high-risk. In-depth data on waning of immune response is needed to assess duration of protection, and evaluation of vaccine-dosage for individuals at risk may be reconsidered. With the data on full-dose (100µg) mRNA-1273evaluation of vaccine-dosage for individuals at risk may be reconsidered, despite the small sample size.

**Disclosures:**

**Jannik Stemler, -**, Ministry of Education and Research (BMBF): Grant/Research Support **Oliver A. Cornely, MD PhD**, DZIF: Advisor/Consultant|DZIF: Board Member|DZIF: Grant/Research Support|DZIF: Honoraria|DZIF: Stocks/Bonds

